# Symptoms of Addictive Eating: What Do Different Health Professions Think?

**DOI:** 10.3390/bs11050060

**Published:** 2021-04-26

**Authors:** Megan Whatnall, Janelle Skinner, Antonio Verdejo-Garcia, Adrian Carter, Robyn M. Brown, Zane B. Andrews, Chris V. Dayas, Charlotte A. Hardman, Natalie Loxton, Priya Sumithran, Tracy Burrows

**Affiliations:** 1 Priority Research Centre for Physical Activity and Nutrition, University of Newcastle, Callaghan, NSW 2308, Australia; megan.whatnall@newcastle.edu.au (M.W.); janelle.skinner@newcastle.edu.au (J.S.); 2 School of Health Sciences, College of Health, Medicine and Wellbeing, University of Newcastle, Callaghan, NSW 2308, Australia; 3 Turner Institute for Brain and Mental Health, Monash University, Clayton, VIC 3800, Australia; antonio.verdejo@monash.edu (A.V.-G.); adrian.carter@monash.edu (A.C.); 4 Florey Institute of Neuroscience and Mental Health, University of Melbourne, Parkville, VIC 3052, Australia; robyn.brown@florey.edu.au (R.M.B.); zane.andrews@monash.edu (Z.B.A.); 5 Monash Biomedicine Discovery Institute and Department of Physiology, Monash University, Clayton, VIC 3800, Australia; 6 School of Biomedical Sciences & Pharmacy, College of Health, Medicine and Wellbeing, University of Newcastle, Callaghan, NSW 2308, Australia; christopher.dayas@newcastle.edu.au; 7 Hunter Medical Research Institute (HMRI), New Lambton Heights, NSW 2305, Australia; 8 Department of Psychology, Institute of Population Health, University of Liverpool, Liverpool L69 7ZA, UK; cah@liverpool.ac.uk; 9 School of Applied Psychology, Griffith University, Brisbane, QLD 4122, Australia; n.loxton@griffith.edu.au; 10 Centre for Youth Substance Abuse Research, University of Queensland, Brisbane, QLD 4072, Australia; 11 Department of Medicine (Austin), University of Melbourne, Heidelberg, VIC 3084, Australia; priyas@unimelb.edu.au; 12 Department of Endocrinology, Austin Health, Heidelberg Heights, VIC 3081, Australia

**Keywords:** addictive eating, food addiction, health professional, clinician

## Abstract

The symptoms of addictive eating are often debated, with some overlap in symptoms with substance addictions or other disorders such as binge eating disorder. This study explored the levels of agreement with symptoms of addictive eating among different health professions, the conditions they provide advice for, and the population group/s they work with. An online cross-sectional survey was conducted in February–April 2020 including 142 health professionals (87% female, 65% residing in Australia, 28% each working in private practice/hospital settings). Of these, 47% were dietitians, 20% psychologists/psychotherapists/counsellors, 16% other health practitioners (e.g., social workers), 13% health researchers, and 5% medical professionals. Agreement with 11 statements relating to addictive eating symptoms was assessed on a scale of 1/strongly disagree to 5/strongly agree (e.g., certain foods produce physiological effects in the brain rewards system). Differences in agreement by health profession were assessed by one-way analysis of variance. There were significant differences in agreement with individual statements between health professions. Psychologists, psychotherapists, and counsellors reported lower agreement to statements relating to physiological effects in the reward system, withdrawal symptoms, and over-eating to alleviate stress/anxiety, than other professions (*p* < 0.05). Those providing advice for disordered eating only reported lower agreement across statements compared with those providing advice for overweight/obesity or both (*p* < 0.001). There were minimal differences based on the population group/s that health professionals work with. There is some agreement among health professionals regarding addictive eating symptoms, however, this differs by profession and the conditions they treat. This study provides a novel perspective on health professionals’ views on addictive eating symptoms, and there is a need for more research to explore the concepts further.

## 1. Introduction

Addictive eating is not currently classified as a distinct eating, substance-related or addictive disorder [1]. However, approximately 15–20% of the population self-report symptoms that align with addictive eating [2], while 66–86% of general community samples endorse the concept of addictive eating [3]. In practice, individuals seeking treatment for addictive eating may present in a number of ways, including weight management, mental health, or disordered eating. Health professionals that may provide treatment to these individuals include but are not limited to, dietitians and nutritionists, psychologists or other mental health care practitioners, and general practitioners. To advance the field of addictive eating, the clinical utility of the proposed disorder needs to be explored [1].

There is an ongoing debate around whether addictive eating should be classified as a distinct disorder, and if so, what the appropriate classification is [1,4,5]. Schulte et al. recently evaluated the evidence for whether addictive eating should be considered as a distinct psychiatric disorder, concluding that it may warrant consideration however further research is necessary [1]. There is also variability and debate in the assessment and conceptualisation of addictive eating. Addictive eating is assessed using various tools, for example, the Yale Food Addiction Scale (YFAS), the Addiction-Like Eating Behaviour Scale, the Loss of Control Over Eating Scale, and the Three-Factor Eating Questionnaire [4,5,6,7,8,9,10]. The YFAS is most commonly used to identify and assess addictive eating [2], which comprises a set of symptoms that map to the diagnostic criteria for substance use disorder, as well as a question on clinical impairment. In systematic reviews, the most common YFAS symptoms reported across studies include persistent desire or unsuccessful attempts to cut down, and continued consumption despite significant adverse consequences (e.g., emotional or physical) [2,11]. The Loss of Control Over Eating Scale, for example, focuses on 12 facets of the eating occasion itself (e.g., increased speed or rate of eating) [12], while others take a broader view of the concept such as the Addiction-Like Eating Behaviour Scale which assesses appetitive drive and low dietary control [10]. There is some cross-over of concepts between the different tools used to assess addictive eating, with differences in the specific focus or perspective and whether they attempt to align with conceptualisations of other disorders, for example, substance-related or eating disorders. Many of the available tools have good convergent validity and reliably conceptualise addictive eating from the perspective of the individual with addictive eating [1,9]. However, there is limited evidence on the views of health professionals on whether they agree with the conceptualised behaviours and symptoms of addictive eating.

Given the debate around the classification and conceptualisation of addictive eating as a disorder, there is also potential confusion among health professionals and therefore it is important to explore their views. This is important considering the potential for different referral or treatment pathways depending on whether different perceptions exist among health professionals. This will also help to understand the potential implication that a recognised diagnosis of addictive eating might have in practice and the clinical utility. Exploring health professionals’ views invites the perspective of those with day-to-day clinical experience and can be useful to inform recommendations for practice. The aim of the current study was to explore the levels of agreement with statements regarding behaviours and symptoms of addictive eating across different health professions.

## 2. Materials and Methods

### 2.1. Study Design

An online cross-sectional survey was conducted from 21 February to 27 April 2020 via Qualtrics (https://www.qualtrics.com/au/, accessed on 8 January 2020). The full survey methods and results are reported elsewhere [13]. Briefly, the aim of the survey was to explore the opinions and understanding of addictive eating behaviours among health professionals. Eligible participants were health professionals (e.g., allied health (professions distinct from nursing and medicine such as dietetics), psychologists, medical professionals (those working in a clinical setting with medical training such as general practitioners)) with experience in the management or research of overweight/obesity or disordered eating. Participants could be from any country; however, the survey was in English.

The survey consisted of 70 questions and took approximately 25 min to complete. The current paper reports on the questions asking participants the degree to which they agreed with statements regarding addictive eating behaviours. These results are reported here separately as they relate more specifically to the clinical utility of addictive eating, whereas the remainder of the questions aimed to gather a more general overview of opinions on addictive eating and preferences for professional development training. Health professionals were recruited via convenience sampling using a range of methods. Email invitations were sent from the research team to their networks, as well as advertisements via Twitter, and the member e-newsletter of Dietitians Australia. The survey was advertised as a “cross-sectional survey to identify the current understanding of addictive eating behaviours and whether a need exists for professional development training”. Ethical approval was obtained from the University of Newcastle Human Research Ethics Committee and informed consent was obtained from all participants prior to participation.

### 2.2. Measures

#### Demographic Characteristics

Data on demographic characteristics were collected including age, gender, country of residence, and highest qualification completed. Participants were asked their occupation (dietitian, psychologist, general practitioner (GP), medical specialist, bariatric surgeon, health practitioner (e.g., nurse, physiotherapist), health researcher, tertiary academic/teacher, other), primary work setting (hospital, primary care, community/population/public health program, private practice, food service, research and teaching, food industry, government, other), the population group/life stage they primarily work with (e.g., adolescents, adults) and whether they provide advice to individuals with overweight/obesity or disordered eating.

### 2.3. Agreement with Statements Regarding Addictive Eating Behaviours and Symptoms

Participants were asked to rate their agreement on a five-point Likert scale from 1/strongly disagree to 5/strongly agree with 11 different statements relating to addictive eating behaviours and symptoms, as listed in the results. The 11 statements were derived by the authors as a consensus of conceptualised addictive eating behaviours and symptoms from key review papers written by experts in the field of addictive eating and the YFAS which is the most commonly used tool to characterise food addiction [4,5,6,7,8,14]. The intention was to phrase the behaviours and symptoms in a neutral light in order to ascertain participants’ views on these items without the potential influence or bias often attributed to the concept of addictive eating [2].

### 2.4. Statistical Analysis

Statistical analysis was conducted using Stata software 14.2 (StataCorp LLC, College Station, TX, USA). Data are reported as means and standard deviations for continuous variables and number and percentage for categorical variables. The level of agreement with statements regarding addictive eating symptoms is reported as mean and standard deviation. Differences in the agreement with statements were examined between health professions, the conditions that they provide advice to clients/individuals for, and the population group/s that they work with, using one-way analysis of variance (ANOVA) with post-hoc Tukey tests. Statistical significance was set at *p* < 0.05. The health profession categories were modified from those asked in the survey to incorporate the responses provided under “other”. The categories for conditions that health professionals provide advice for and population group/s that they work with were modified so that each respondent was unique, as these were initially asked as multiple response questions. The modified categories are those reported in Table 1. The proportion of participants agreeing/disagreeing with each statement by health profession is available as Supplementary Material (Table S1).

## 3. Results

### 3.1. Sample Characteristics

In total, 142 health professionals completed the survey. The mean ± SD age of participants was 40.7 ± 11.7 years, and most were female (*n* = 123, 87%) (Table 1). Participants were largely from Australia (*n* = 92, 65%) or the USA (*n* = 23, 16%). The most common professions were dietitian (*n* = 66, 47%) and psychologist, psychotherapist, or counsellor (*n* = 23, 20%), followed by other health practitioner (e.g., nurse, physiotherapist, social worker) (*n* = 23, 16%). All health professionals had experience with overweight and obesity through practice, research, and/or tertiary teaching. Among the sample, 54% (*n* = 76) reported that they directly provide advice to clients for disordered eating and overweight/obesity, while 10% (*n* = 14) provide advice for disordered eating only, and 17% (*n* = 24) for overweight/obesity only. Of the sample, 11% (*n* = 15) reported that the population group/s they work with include infants, children, and/or adolescents, while 67% (*n* = 92) work with young adults, adults, and/or older adults, and 23% (*n* = 31) work with individuals across the lifespan.

### 3.2. Agreement with Statements Regarding Addictive Eating Behaviours and Symptoms

Across the 11 statements, the mean ± SD agreement ranged from 3.5 ± 1.3 to 4.5 ± 0.9 (maximum/5 = strongly agree). Overall, the mean ± SD agreement was ≥4 for seven of the 11 statements, corresponding to agree/strongly agree responses (Table 2). The statement with the highest agreement was “People can overeat more foods when experiencing stress, anxiety or negative experiences (i.e., comfort eating)”, and the statement with the lowest agreement was “People can have an increased tolerance of foods that are regularly over consumed without experiencing any satiety effects”.

Overall, the highest agreement (i.e., most support for symptoms occurring) was reported among GPs, medical specialists, and medical registrars (specialists-in-training) (4.5 ± 0.4), while the lowest agreement was reported among psychologists, psychotherapists, and counsellors (3.7 ± 1.0), however the difference between professions was not significant (*p* = 0.05). There were significant differences between professions for three of the 11 statements (*p* < 0.05). Generally, psychologists, psychotherapists, and counsellors reported lower agreement to statements relating to physiological effects in the rewards system, withdrawal symptoms, and over-eating to alleviate stress and anxiety, than other professions.

Comparing agreement by the conditions that health professionals provide advice to clients for, those reporting that they provide advice for disordered eating only had significantly lower agreement (average across statements: 3.1 ± 0.7) than those providing advice for overweight/obesity only (4.5 ± 0.4), both conditions (4.0 ± 0.9) or those that selected neither condition (4.4 ± 0.5) (*p* < 0.001). There were significant differences across these categories for eight of the 11 statements (Supplementary Table S2). Generally, those reporting that they provide advice for disordered eating only reported lower agreement to statements relating to physiological effects in the rewards system, continued overconsumption despite health risks, increased tolerance to overconsumed foods, withdrawal symptoms, cravings to particular foods, exhibiting addiction-like behaviour, and over-eating to alleviate stress and anxiety, than those providing advice for overweight/obesity only, both, and those that selected neither condition.

Comparing agreement by the population group/s that health professionals work with, the lowest agreement was among those who work with individuals across the lifespan (3.9 ± 0.9) and the highest among those that work with infants, children, and/or adolescents (4.3 ± 0.4); however, the difference was not significant (*p* > 0.05) (Supplementary Table S3). There was a significant difference for one of the 11 statements, including the lowest agreement among those who work with individuals across the lifespan (3.1 ± 1.2) and highest among those working with infants, children, and/or adolescents (4.1 ± 0.8) for “People can have an increased tolerance of foods that are regularly over consumed without experiencing any satiety effects”.

## 4. Discussion

This survey found that the majority of health professionals agreed with each of the proposed addictive eating behaviours and symptoms. This provides an interesting perspective, as when asked whether addictive eating exists as a disorder, just over half of the sample (60%) supported the concept [13]. This suggests that health professionals are more willing to recognise addictive eating symptomatology than to recognise addictive eating as a disorder. There is also disagreement among researchers in defining addictive eating, the terminology used, and the symptoms [1,5,11]. This survey is the first to explore health professionals’ views on addictive eating. While this provides a novel perspective, the literature for comparison is limited.

Differences in agreement were identified between health professions. GPs, medical specialists, and medical registrars had the highest agreement and psychologists, psychotherapists, and counsellors the lowest across the predefined symptoms. Differences in agreement were also identified between health professionals who reported providing advice to individuals for disordered eating, overweight/obesity, or both. Those providing advice to individuals for disordered eating only reported the lowest agreement across symptoms, and those providing advice to individuals for overweight/obesity only reported the highest agreement. Given that these differences exist, it is also likely that differences exist in the treatment that individuals presenting with addictive eating receive depending on the health professional. This includes which condition/s the health professional typically consults for, and the reason for referral of the individual. This might also explain why many individuals seeking help for addictive eating report that they have seen a range of health professionals. There were minimal differences however between health professionals based on whether they work with infants, children and adolescents, adults, or across the lifespan. This lack of difference is interesting, given that studies have demonstrated the severity of addictive eating is often greater with increased age [15,16,17]. This might have meant greater agreement among those working with adults; however, the lack of difference could be due to the small proportion of the sample who work with infants, children and/or adolescents only in this study (11%) or given the period of adolescence and the wide variety of changes that occur or are reported at this age.

In terms of the addictive eating symptoms, there was clear support (i.e., mean agreement of ≥4/5) for seven of the 11 statements. The highest support was for overconsumption of foods in times of stress, anxiety, or negative experiences (i.e., comfort eating), and repeated attempts to give up particular foods with numerous unsuccessful attempts. The lowest support was for individuals having an increased tolerance to overconsumed foods without satiety, and experiencing withdrawal symptoms (e.g., irritability, headaches, dizziness) when trying to give up some foods. This coincides with the views of community samples, where it was found that food-related tolerance and physical withdrawal symptoms were not regarded as necessary characteristics of addictive eating [18]. The lower support for tolerance to overconsumed foods and withdrawal symptoms could be due to the limited research of these symptoms in human studies and the lack of clear definitions. To facilitate research in this area, Schulte et al. developed the Highly Processed Food Withdrawal Scale to operationalise withdrawal symptoms that may occur during attempts to cut down on highly processed foods [1].

This study provides a novel perspective on health professionals’ views on addictive eating. In particular, exploring the differences between different health professions is important in terms of understanding the treatment or lack of that they may provide or refer to for individuals reporting addictive eating behaviours and symptoms. A limitation of this work is that the health professionals were not asked their views on the question of clinical impairment relating to addictive eating, which is often used to determine whether an individual has addictive eating or not. Additionally, the sample was predominantly female, and dietitians or psychologists, which may limit the scope of views and the generalisability of the findings. In particular, the sample included a small proportion of GP’s, medical specialists, and medical registrars. As these health professionals may be the first point of consultation and the primary health professional to provide a referral for further treatment, their views are important to explore. For this reason, these health professionals were considered as their own category within this study, despite being small in number. This limitation should be considered when interpreting the study findings, and further study is needed to explore the views of GP’s and medical specialists. This study did not consider the level of experience of health professionals, for example, the number of years practicing. This may also be an important consideration in future work.

The key implications of this work for practice include the need:

- To increase awareness of addictive eating among all health professionals as individuals with symptoms of addictive eating may seek assistance from a range of health professionals. This includes awareness of the possible symptoms of and treatment/referral options for addictive eating.
- For health professionals to be aware of the range of terminology used to describe addictive eating, and that this may differ between different health professionals and individuals in the community/individuals seeking treatment.

## 5. Conclusions

Overall, this survey identified that there is agreement among health professionals regarding addictive eating symptoms. However, this differs by profession and the conditions that they typically see clients/individuals for. The key implications from this study are the importance of ensuring there is awareness around addictive eating among health professionals, including awareness of different perspectives, and that there is a need for more research among health professionals to explore the concepts around addictive eating further.

## Figures and Tables

**Table 1:** Demographic characteristics of health professionals participating in a survey on addictive eating (*n* = 142).

Characteristic	*N*	%
**Age (years) Mean ± SD**	40.7 ± 11.7	
**Gender**		
Female	123	86.6
Male	17	12.0
Other	2	1.4
**Country of residence**		
Australia	92	64.8
USA	23	16.2
UK	16	11.3
Canada	9	6.3
New Zealand	1	0.7
Belgium	1	0.7
**Highest qualification completed**		
School certificate/Higher school certificate	2	1.4
Trade or diploma	1	0.7
Undergraduate university degree	39	27.5
Postgraduate university degree	69	48.6
Higher research degree	31	21.8
**Occupation**		
Dietitian	66	46.5
Psychologist/Psychotherapist/Counsellor	28	19.7
Other health practitioner (e.g., nurse, physiotherapist, social worker)	23	16.2
Health researcher/Tertiary academic or teacher	18	12.7
General Practitioner/Medical Specialist/Registrar	7	4.9
**Primary work situation**		
Hospital	39	27.5
Private practice	39	27.5
Research and teaching	29	20.4
Community/population/public health program	19	13.4
Primary care	7	4.9
Food service	1	0.7
Other	8	5.6
**Population group work with**		
Infants (<2 years), children (2–12 years), and/or adolescents (13–17 years)	15	10.9
Young adults (18–24 years), adults (25–65 years), and/or older adults (>65 years)	92	66.7
Across the lifespan	31	22.5
Not applicable	4	2.8
**Conditions provide advice to clients/individuals for**		
Disordered eating	14	9.9
Overweight/obesity	24	16.9
Disordered eating and overweight/obesity	76	53.5
Neither disordered eating or overweight/obesity	16	11.3
Not applicable	12	8.5

**Table 2:** Agreement with addictive eating symptoms by health profession.

Addictive Eating Symptom	Agreement (Mean ± SD)					
	Dietitian (*n* = 66)	Psychologist/Psychotherapist/Counsellor (*n* = 28)	Other Health Practitioner (*n* = 23)	Health Researcher or Academic (*n* =18)	GP/Medical Specialist/Medical Registrar (*n* = 7)	Total (*n* = 142)
Certain foods produce physiological effects in the brain rewards system ^a^	4.2 ± 1.0 ^b^	3.5 ± 1.4 ^b,c,d,e^	4.3 ± 0.9 ^c^	4.4 ± 0.6 ^d^	4.9 ± 0.4 ^e^	4.1 ± 1.1
People repeatedly try to give up particular foods with many unsuccessful attempts	4.4 ± 0.7	4.3 ± 1.0	4.2 ± 1.0	4.4 ± 1.0	4.4 ± 0.5	4.4 ± 0.9
People can continue eating certain foods even when that causes family or work problems	3.9 ± 1.2	3.9 ± 1.3	3.8 ± 1.4	4.0 ± 1.3	3.9 ± 0.9	3.9 ± 1.2
People continue to over consume food despite the increased risk of adverse health consequences	4.2 ± 1.2	3.5 ± 1.7	4.4 ± 1.2	4.4 ± 1.1	4.9 ± 0.4	4.2 ± 1.3
People can have an increased tolerance of foods that are regularly over consumed without experiencing any satiety effects	3.6 ± 1.2	3.0 ± 1.5	3.7 ± 1.1	3.4 ± 1.4	4.1 ± 0.7	3.5 ± 1.3
People exhibit withdrawal symptoms (e.g., irritability, headaches, dizziness) when trying to give up some foods ^a^	3.4 ± 1.2 ^b^	3.1 ± 1.4 ^c^	4.3 ± 1.1 ^b, c^	3.8 ± 1.2	3.8 ± 0.9	3.6 ± 1.3
People over consume food in excessive amounts	3.9 ± 1.3	3.4 ± 1.6	4.3 ± 1.0	4.3 ± 1.0	4.7 ± 0.5	4.0 ± 1.3
People exhibit/report strong cravings or desire to consume particular foods or food types	4.3 ± 0.8	4.0 ± 1.2	4.4 ± 1.0	4.7 ± 0.5	4.7 ± 0.5	4.3 ± 0.9
People can exhibit associations with food and food behaviours that could be likened to an addiction	3.9 ± 1.2	3.2 ± 1.6	4.1 ± 1.3	4.2 ± 1.1	4.6 ± 0.8	3.9 ± 1.3
People can exhibit associations with food and food behaviours that impact on their daily functioning	4.2 ± 0.8	4.3 ± 1.0	4.4 ± 0.9	4.1 ± 1.2	4.7 ± 0.5	4.2 ± 0.9
People can overeat more foods when experiencing stress, anxiety or negative experiences (i.e., comfort eating) ^a^	4.5 ± 0.7	4.0 ± 1.2 ^b^	4.6 ± 0.9	4.8 ± 0.4 ^b^	5.0 ± 0.0	4.5 ± 0.9
**Average agreement**	**4.1 ± 0.8**	**3.7 ± 1.0**	**4.2 ± 0.9**	**4.2 ± 0.8**	**4.5 ± 0.4**	**4.1 ± 0.9**

^a^ Indicates statistically significant difference overall between professions assessed via one-way analysis of variance (*p* < 0.05). ^b,c,d,e^ Cells with the same superscript letter indicates statistically significant difference between groups assessed via post-hoc Tukey test (*p* < 0.05).

## Supplementary Materials

The following are available online at https://www.mdpi.com/article/10.3390/bs11050060/s1, Table S1: Agreement with addictive eating symptoms by health profession (%), Table S2: Agreement with addictive eating symptoms by conditions that health professionals provide advice to clients/individuals for, Table S3: Agreement with addictive eating symptoms by population group/s that health professionals work with.
