# Supplementary material for: Symptoms of Addictive Eating: What Do Different Health Professions Think?
Source: Behav Sci (Basel). 2021 Apr 26;11(5):60. doi: 10.3390/bs11050060 (PMC8145409; doi:10.3390/bs11050060)
Supplement: Supplementary file 1 [file behavsci-11-00060-s001.zip › supplementary.pdf]

Table S1 Agreement with addictive eating symptoms by health profession (%)

Statement	Agree/Disagree	Dietitian (n=66)	Psychologist/ Psychotherapist/ Counsellor (n=28)	Other health practitioner (n=23)	Health researcher or academic (n=18)	GP /Medical Specialist/ Medical Registrar (n=7)	Total (n=142)
Certain foods produce physiological effects in the brain rewards system	Strongly agree	40.9	28.6	47.8	50.0	85.7	43.0
	Agree	45.5	28.6	43.5	44.4	14.3	40.1
	Neutral	7.6	17.9	4.4	5.6	0.0	8.5
	Disagree	1.5	10.7	0.0	0.0	0.0	2.8
	Strongly disagree	4.6	14.3	4.4	0.0	0.0	5.6
People repeatedly try to give up particular foods with many unsuccessful attempts	Strongly agree	51.5	53.6	47.8	61.1	42.9	52.1
	Agree	43.9	35.7	39.1	27.8	57.1	40.1
	Neutral	1.5	3.6	4.4	5.6	0.0	2.8
	Disagree	1.5	3.6	4.4	0.0	0.0	2.1
	Strongly disagree	1.5	3.6	4.4	5.6	0.0	2.8
People can continue eating certain foods even when that causes family or work problems	Strongly agree	39.4	46.4	43.5	50.0	28.6	42.3
	Agree	30.3	21.4	21.7	22.2	28.6	26.1
	Neutral	18.2	17.9	17.4	16.7	42.9	19.0
	Disagree	6.1	7.1	4.4	0.0	0.0	4.9
	Strongly disagree	6.1	7.1	13.0	11.1	0.0	7.8
People continue to over consume food despite the increased risk of adverse health consequences	Strongly agree	59.1	50.0	65.2	66.7	85.7	60.6
	Agree	22.7	7.1	26.1	16.7	14.3	19.0
	Neutral	9.1	14.3	0.0	11.1	0.0	8.5
	Disagree	1.5	3.6	0.0	0.0	0.0	1.4
	Strongly disagree	7.6	25.0	8.7	5.6	0.0	10.6
People can have an increased tolerance of foods that are	Strongly agree	27.3	21.4	26.1	33.3	28.6	26.8
	Agree	37.9	17.9	34.8	11.1	57.1	31.0

regularly over consumed without experiencing any satiety effects	Neutral	15.2	25.0	26.1	27.8	14.3	20.4
	Disagree	10.6	14.3	8.7	16.7	0.0	11.3
	Strongly disagree	9.1	21.4	4.4	11.1	0.0	10.6
People exhibit withdrawal symptoms (e.g. irritability, headaches, dizziness) when trying to give up some foods	Strongly agree	15.2	14.3	65.2	33.3	28.6	26.1
	Agree	43.9	35.7	13.0	38.9	28.6	35.9
	Neutral	21.2	17.9	13.0	11.1	42.9	19.0
	Disagree	7.6	10.7	4.4	11.1	0.0	7.8
	Strongly disagree	12.1	21.4	4.4	5.6	0.0	11.3
People over consume food in excessive amounts	Strongly agree	40.9	39.3	52.2	55.6	71.4	45.8
	Agree	37.9	14.3	34.8	33.3	28.6	31.7
	Neutral	6.1	17.9	8.7	5.6	0.0	8.5
	Disagree	4.6	7.1	0.0	0.0	0.0	3.5
	Strongly disagree	10.6	21.4	4.4	5.6	0.0	10.6
People exhibit/report strong cravings or desire to consume particular foods or food types	Strongly agree	48.5	42.9	60.9	66.7	71.4	52.8
	Agree	42.4	32.1	26.1	33.3	28.6	35.9
	Neutral	6.1	14.3	8.7	0.0	0.0	7.0
	Disagree	1.5	3.6	0.0	0.0	0.0	1.4
	Strongly disagree	1.5	7.1	4.4	0.0	0.0	2.8
People can exhibit associations with food and food behaviours that could be likened to an addiction	Strongly agree	36.4	28.6	56.5	50.0	71.4	41.6
	Agree	39.4	21.4	21.7	27.8	14.3	30.3
	Neutral	7.6	17.9	8.7	16.7	14.3	11.3
	Disagree	9.1	3.6	4.4	0.0	0.0	5.6
	Strongly disagree	7.6	28.6	8.7	5.6	0.0	11.3
People can exhibit associations with food and food behaviours that impact on their daily functioning	Strongly agree	31.8	53.6	56.5	50.0	71.4	44.4
	Agree	59.1	25.0	39.1	27.8	28.6	43.7
	Neutral	6.1	17.9	0.0	11.1	0.0	7.8
	Disagree	0.0	0.0	0.0	5.6	0.0	0.7

	Strongly disagree	3.0	3.6	4.4	5.6	0.0	3.5
People can overeat more foods when experiencing stress, anxiety or negative experiences (i.e. comfort eating)	Strongly agree	59.1	50.0	73.9	83.3	100.0	64.8
	Agree	34.9	21.4	17.4	16.7	0.0	25.4
	Neutral	4.6	17.9	4.4	0.0	0.0	6.3
	Disagree	0.0	3.6	0.0	0.0	0.0	0.7
	Strongly disagree	1.5	7.1	4.4	0.0	0.0	2.8

Table S2 Agreement with addictive eating symptoms by conditions that health professionals provide advice to clients/individuals for

Addictive eating symptom	Agreement (Mean±SD)			
	Disordered eating (n=14)	Overweight/ obesity (n=24)	Both (n=76)	Neither (n=16)
Certain foods produce physiological effects in the brain rewards system [a]	3.2±1.4 [b, c, d]	4.6±0.6 [b]	4.0±1.1 [c]	4.3±0.6 [d]
People repeatedly try to give up particular foods with many unsuccessful attempts	4.1±1.4	4.5±0.7	4.4±0.9	4.5±0.6
People can continue eating certain foods even when that causes family or work problems	3.3±1.5	4.0±1.3	3.9±1.2	4.3±0.8
People continue to over consume food despite the increased risk of adverse health consequences [a]	2.6±1.5 [b, c, d]	4.8±0.4 [b]	4.1±1.4 [c]	4.7±0.5 [d]
People can have an increased tolerance of foods that are regularly over consumed without experiencing any satiety effects [a]	2.2±1.2 [b, c, d]	4.2±0.8 [b]	3.5±1.3 [c]	4.2±0.8 [d]
People exhibit withdrawal symptoms (e.g. irritability, headaches, dizziness) when trying to give up some foods [a]	2.2±1.1 [b, c, d]	4.1±0.8 [b, e]	3.4±1.3 [c, e, f]	4.4±0.7 [d, f]
People over consume food in excessive amounts [a]	2.9±1.5 [b, c, d]	4.6±0.5 [b, e]	3.8±1.4 [c, e,]	4.6±0.7 [d]
People exhibit/report strong cravings or desire to consume particular foods or food types [a]	3.9±1.0 [b]	4.7±0.5 [b]	4.2±1.0	4.5±0.7
People can exhibit associations with food and food behaviours that could be likened to an addiction [a]	2.5±1.4 [b, c, d]	4.5±0.7 [b, e]	3.7±1.4 [c, e,]	4.5±0.5 [d]
People can exhibit associations with food and food behaviours that impact on their daily functioning	3.9±1.2	4.5±0.5	4.2±1.0	4.4±0.7
People can overeat more foods when experiencing stress, anxiety or negative experiences (i.e. comfort eating) [a]	3.9±0.9 [b]	4.8±0.4 [b]	4.4±1.0	4.6±0.5
**Average agreement** [a]	**3.1±0.7** [b, c, d]	**4.5±0.4** [b, e]	**4.0±0.9** [c, e,]	**4.4±0.5** [d]

N=12 indicated not applicable, therefore this is out of 130 respondents. [a] Indicates statistically significant difference overall assessed via one-way analysis of variance (p<0.05). [b, c, d, e] Cells with the same superscript letter indicates statistically significant difference between groups assessed via post-hoc Tukey test (p<0.05).

Table S3 Agreement with addictive eating symptoms by population group/s that health professionals work with

Addictive eating symptom	Agreement (Mean±SD)		
	Infants, children and/or adolescents (n=15)	Young adults, adults and/or older adults (n=92)	Across the lifespan (n=31)
Certain foods produce physiological effects in the brain rewards system	4.4±0.5	4.2±1.1	3.9±1.3
People repeatedly try to give up particular foods with many unsuccessful attempts	4.6±0.5	4.3±0.9	4.4±1.0
People can continue eating certain foods even when that causes family or work problems	3.7±1.2	3.8±1.3	4.2±1.0
People continue to over consume food despite the increased risk of adverse health consequences	4.7±0.6	4.2±1.3	3.9±1.4
People can have an increased tolerance of foods that are regularly over consumed without experiencing any satiety effects [a]	4.1±0.8 [b]	3.6±1.3	3.1±1.2 [b]
People exhibit withdrawal symptoms (e.g. irritability, headaches, dizziness) when trying to give up some foods	4.1±0.7	3.6±.1.3	3.4±1.3
People over consume food in excessive amounts	4.2±0.7	4.0±1.4	3.9±1.3
People exhibit/report strong cravings or desire to consume particular foods or food types	4.5±0.6	4.3±0.9	4.3±1.0
People can exhibit associations with food and food behaviours that could be likened to an addiction	4.1±0.7	4.0±1.2	3.5±1.6
People can exhibit associations with food and food behaviours that impact on their daily functioning	4.4±0.6	4.3±0.9	4.1±1.0
People can overeat more foods when experiencing stress, anxiety or negative experiences (i.e. comfort eating)	4.8±0.4	4.5±0.9	4.3±1.0
**Average agreement**	**4.3±0.4**	**4.1±0.9**	**3.9±0.9**

N=4 indicated not applicable, therefore this is out of 138 respondents. [a] Indicates statistically significant difference overall between population groups that health professionals work with assessed via one-way analysis of variance ($p<0.05$). [b, c, d, e] Cells with the same superscript letter indicates statistically significant difference between groups assessed via post-hoc Tukey test ($p<0.05$).
